# Scaling up healthy eating in early childhood education and care: evaluation of the Appetite to Play capacity-building intervention

**DOI:** 10.1017/S1368980024001290

**Published:** 2024-09-16

**Authors:** E Jean Buckler, Kasra Hassani, Jennifer McConnell-Nzunga, Sana Fakih, Jennifer Scarr, Louise C Mâsse, Patti-Jean Naylor

**Affiliations:** 1 School of Exercise Science, Physical and Health Education, Faculty of Education, University of Victoria, Victoria, BC V8W 2Y2, Canada; 2 Child Health BC, Provincial Health Services Authority, Vancouver, BC V6J 4YC, Canada; 3 School of Population and Public Health, Faculty of Medicine, University of British Columbia, Vancouver, BC V6T 1Z3, Canada; 4 British Columbia Children’s Hospital Research Institute, 4480 Oak St., Vancouver, BC V6H 3N1, Canada

**Keywords:** Nutrition, Early childhood education, Professional development, Healthy eating, Scale up

## Abstract

**Objective:**

The purpose of this study was to examine the dissemination of the healthy eating component of Appetite to Play at scale using the Reach, Effectiveness, Adoption, Implementation, Maintenance (RE-AIM) framework.

**Design:**

The Appetite to Play capacity-building intervention is a set of evidence-informed implementation strategies aimed at enhancing the adoption of recommended practices for promoting healthy eating and active play in early years settings. The evaluation was pragmatic, employing both quantitative (surveys) and qualitative (interviews) data collection.

**Setting:**

The Appetite to Play intervention was delivered through in-person community-based workshops, virtual workshops, asynchronous e-learning and online resources.

**Participants:**

We received completed surveys from 1670 in-person workshop participants (96 % female), and twenty-three (all female) survey respondents also participated in a telephone interview. Approximately two-thirds of all participant groups were certified early childhood educators.

**Results:**

Results indicated that Appetite to Play had high reach (25 867 individual website visits, 195 workshops delivered), effectiveness (significant increases in care provider’s knowledge, confidence (*P* < 0·05) and high post-intervention intention to implement), adoption (11 % of educators in BC trained) and implementation (good alignment with implementation strategies and current practices), with a significant maintenance plan to support the intervention’s future success.

**Conclusions:**

An evidence-based capacity-building intervention with an emphasis on training and provision of practical online resources can improve early years providers’ knowledge, confidence and intention to implement recommended practices that promote healthy eating. Further research is needed to determine the impact on child-level outcomes and how parents can be supported in contributing to positive food environments.

Early childhood, particularly the preschool years (3–5 years), is an important time for the development of healthy eating behaviours. Healthy eating behaviours play a key role in health throughout the lifespan, with a healthy diet associated with reduced risks of all-cause mortality^(1,2)^. Food preferences in young children are greatly influenced by familiarity with foods, indicating that the food environment plays a key role in eating behaviours^(1)^. With an increasing number of Canadian children attending early childhood education and care (ECEC) settings, the ECEC environment is receiving recognition as an important influence on childhood eating behaviours. There is evidence to suggest that ECEC, along with the home environment, influences the eating behaviours of young children^(3)^ and unfortunately that foods served in ECEC are of insufficient nutritional quality^(4)^.

Strategies to improve the food environment in ECEC have had some positive results^(5,6)^. For example, small, but significant increases in fruits and vegetables consumed were documented in Healthy Start-Départ Santé, a recent Canadian intervention in childcare centres^(7)^. Results from a systematic and an umbrella review indicate that multi-level, capacity-building interventions are more likely to be successful at improving food environments and behaviours^(5,6)^. This is also supported by evidence from the implementation science literature^(8)^ One example of a capacity-building strategy is training and Matwiejczyk and colleagues’ umbrella review highlighted that several systematic reviews they analysed called for the training of early years practitioners^(5)^.

In the health promotion field, capacity building is a practice where skills, knowledge, structure and systems are developed at multiple levels to support change in practices and at the individual and organisational level^(9)^. Capacity building has been referred to by implementation scientists as an implementation strategy category, including implementation strategies such as the provision of training, technical assistance and tools or resources to support implementation by delivery system providers^(8)^. Capacity-building interventions are typically delivered by support system providers (e.g. public health nutritionists or technical support units) that serve delivery system providers (e.g. early years childcare providers or school teachers)^(10)^. Capacity-building outcomes are typically at the delivery system level and include self-efficacy and motivation to implement as well as measures of implementation^(8)^. However, health interventions typically take place on a small scale, with minimal plans for scale-up or population-level implementation^(11)^. In implementation science, capacity-building strategies like training and the provision of tools and resources that are used for the scale-up of specific evidence-based interventions are also defined as scale-up strategies^(8)^.

In British Columbia, Canada, a capacity-building intervention (Appetite to Play) was developed to build the knowledge, confidence and intentions of early years care providers to incorporate recommended practices for promoting healthy eating behaviours, as well as physical activity and physical literacy in childcare settings. Appetite to Play can best be described as a bundle of implementation strategies to support adoption and implementation of those evidence-based recommended practices^(10,12–18)^. Appetite to Play was developed for scale-up at the population level using empirical evidence from other jurisdictions and partner input, including researchers and practitioners. This is in contrast to traditionally researcher-driven pathways where formative and pilot testing work is followed by efficacy testing and then larger randomised controlled trials in real world conditions to test effectiveness or effectiveness at scale^(11,19)^. The capacity-building intervention was developed in response to updates to government-level policies released in 2016 and enforced in 2017, to support ECEC professionals in implementing new active play policies and enhancing their implementation of previously established healthy eating policies. Thus, we developed the evaluation strategies to document the effectiveness of this real-world trial. Appetite to Play can be categorised as a practice-based evidence pathway, which Ogilvie and colleagues indicate should be integrated into research evidence^(11)^. Indig and colleagues would categorise Appetite to Play as a Type IV scale-up as the intervention was disseminated at scale without further pilot and efficacy trials^(19)^. Results of the capacity-building intervention physical activity and physical literacy components were previously reported on^(20)^.

## Methods

Appetite to Play was developed in line with evidence-based recommendations and strategies from the early years and implementation science and public health capacity-building literature^(10,12–18)^. Content was delivered via in person or online training workshops, a website based toolkit of resources, technical support, a community of practice and marketing and communication tools; details of these intervention components were outlined in a previous publication^(20)^. These capacity-building activities were aligned with relevant literature^(15,16)^ including Wandersman and colleagues’ Getting to Outcomes Framework^(13)^, Powell and colleagues Expert Recommendations for Implementing Change^(21)^ and Protcor and colleagues Specifying and Reporting of Implementation Strategies^(22)^. Table 1 provides detail on the specification of the implementation strategies comprising the Appetite to Play bundle aligned with Expert Recommendations for Reporting Change^(21)^ and Specifications and Reporting of Implementation Strategies, and the content of the training and resources is described following.

**Table 1:** Healthy eating capacity-building implementation strategy components of Appetite to Play aligned with expert recommendations for implementing change (ERIC)^(17)^ and specifying and reporting of implementation strategies^(22)^

Implementation Strategy Component	Description*	Alignment with ERIC Taxonomy
Development of both recommended practices and capacity-building implementation strategy bundle	The intervention was developed (Action) by Child Health BC in consultation with early years partners (Actors) throughout the region in order to meet the needs of early years providers (Target) who would, after exposure to the capacity-building, enhance their practices and the provision of opportunities for healthy eating and active play (Action) in the early years environment (Context). An advisory group, including representatives from local health authorities, provincial health and childcare ministries, federal public health agencies, childcare organisations and relevant health-related non-profits, was established. Research team members also sat in on the consultation and advisory groups. Funding came from a suite of resources, including provincial and federal funding bodies.	Access new funding, Assess for readiness and identify barriers and facilitators; Build a coalition; Conduct local consensus discussions; Develop academic partnerships; Identify early adopters; Inform local leaders; Involve patients/consumers; Obtain and use consumer feedback; Stage implementation scale up; Use advisory boards and workgroups
Training	ATP training facilitators were trained (Action) by an expert ATP Central Support Unit consultant (Actor): a dietitian with expertise in both the healthy eating content and facilitation. Training workshops were then delivered in-person (action) in local communities (context) by trained ATP facilitators (actors) to early years providers (target) Training options and dose were: (1) a 3 h in-person workshop in a community setting (context) and eligible for three professional development (PD) credits (1·5 h was dedicated to eating and food literacy), or (2) a 3 h live-online workshop (context) delivered by Appetite to Play Central Support expert consultant via GoToMeeting (LogMeIn Inc, Boston, MA, USA) eligible for 3 PD credits similar to in-person workshops, or (3) a self-paced e-learning module in food literacy, eligible for 1·5 h of PD credits.	Alter incentives; Conduct ongoing training; Make training dynamic; alter incentives; Use train-the-trainer strategies
Toolkit (tools)	A website based early years provider ‘toolkit’ (www.appetitetoplay.com (context)) was developed and managed by the Appetite to Play Central Technical Support Unit and consultants (actors). The website, housed (1) a set of interactive tools to assist early years providers in program planning (e.g. weekly meal planner, and self-assessment/audit tool), (2) a set of healthy eating recommended practices, (3) food related activity ideas, recipes, tips and direction, (4) access to the e-learning modules and (5) registration for online workshops. Marketing and communications materials and/or training introduced early years providers to the website/toolkit.	Develop and implement tools for quality monitoring; Distribute educational materials
Technical support	After training, early years providers (target) had access to regular new content developed by the ATP Central Technical Support Unit (actor) including new recipes, tips and activity ideas via social media, e-newsletters and website updates to support them taking action on the recommended practices; email and telephone support was also provided for users inquiring and registering for training	Centralize technical assistance; Provide ongoing consultation
Community of practice	A community of practice was piloted by the Appetite to Play Central Technical Support Unit (actor) in collaboration with British Columbia (BC) Early Years Professional Development (EYPD) portal. EYPD is a hub for early years professionals to find and post training events for PD. The forum was moderated by regional trainers and monitored by the Appetite to Play Central Technical Support team and lead trainers.	Capture and share local knowledge; Create a learning collaborative; Promote network weaving
Marketing and communications	Postcards, brochures, branded giveaways and a promotional video were created by the Appetite to Play Central Technical Support Unit (actor) and used to promote (action) Appetite to Play to early years providers (target) across the province through professional organizations e-mail/newsletter distribution, conferences and local Childcare Resource and Referral agencies. Licensing officers also promoted the training and resources to early years providers as a tool that could be used to meet licensing requirements. Weekly social media posts and bimonthly e-newsletters were also used to promote the initiative.	Distribute educational materials; use mass media

Table partially adapted and reproduced with permission from: Hassani K, Buckler EJ, McConnell-Nzunga J, Fakih S, Scarr J, Mâsse LC, Naylor PJ. Implementing Appetite to Play at scale in British Columbia: Evaluation of a Capacity-Building Intervention to Promote Physical Activity in the Early Years. International Journal of Environmental Research and Public Health. 2020 Jan;17(4):1132^(10)^.

* Description enhanced to adhere to guidelines for specifying and reporting of implementation strategies. (Proctor, E.K., et al. (2013).

In person and virtual (synchronous) online training workshops that took place in 2017–2019 provided both healthy eating and physical activity content, with approximately half of the time focusing on each content area. Asynchronous e-learning modules focusing only on healthy eating were delivered and the website, which served as a toolkit of online and printable resources had healthy eating focused sections (recipes, menu planning, activities and recommended practices). Training was a core implementation strategy and the content of it and the toolkit focused on supporting nine recommended practices for food and feeding in ECEC: (1) offer a variety of foods from Canada’s Food Guide for meals and snacks; (2) make water available throughout the day; (3) support infant feeding; (4) support children to become good eaters; (5) offer safe food and beverages; (6) create a physical space that supports healthy eating; (7) educate staff to model and promote positive habits; (8) communicate regularly with families and share information about food and healthy eating and (9) develop policies for food and feeding^(23)^. These recommended practices were developed based on empirical evidence and through consultation with an advisory group^(14,17,18,24,25)^. All three training modalities delivered similar content, with identical content delivered via the in person and virtual workshops, and a more focused discussion of healthy eating and food literacy in the e-learning modules.

As previously outlined in our physical activity paper^(20)^, Appetite to Play, the capacity-building intervention, was developed for dissemination at scale in consultation and engagement with early years partners throughout the dissemination region. Through this process, the capacity-building intervention was built to meet the needs of early years providers and partner expertise and experience informed the dissemination plan. Child Health BC led the development and dissemination, and acted as Appetite to Play Central Support, implemented in partnership with three other non-profit organisations: the YMCA of Greater Vancouver, Childhood Obesity Foundation and Sport for Life, as well as researchers at the University of Victoria and University of British Columbia. Over time, the British Columbia Recreation and Parks Association became another key delivery partner. An advisory group of fourteen individuals, with membership from relevant provincial and municipal government employees, community nutritionists, physical activity, sport and ECEC professionals, was set up by Child Health BC. The advisory group met approximately 2 times/year over 4 years to advise on the development of both the resource, dissemination strategy, implementation (course correction) and sustainability framework.

Details of the dissemination at scale and implementation strategy have been outlined in a previous publication focused on the physical activity component^(20)^. Training was a core strategy so we briefly overview this, with specific details about the healthy eating content training. Appetite to Play employed a ‘train the trainer’ model to deliver the intervention throughout the region of interest^(26,27)^. Healthy eating content was developed by registered dietitian nutrition consultants with relevant content expertise in the topics. Two lead trainers, one expert in physical activity and one in nutrition, delivered both the healthy eating and physical activity content, and these experts trained regional trainers from widely dispersed geographic regions (*n* 88) to deliver the workshop. Regional trainers were required to possess a diploma or degree in a relevant field (e.g. early childhood education, nutrition, kinesiology, recreation and education). Ongoing support, in the form of two refresher training sessions, additional support with a lead trainer upon request, follow-up support if a negative review was received regarding a regional trainer and a newsletter and cross-site sharing, was provided to regional trainers. Finally, a regular newsletter and cross-site sharing were put in place to support regional trainers.

### Evaluation design and framework

The evaluation of Appetite to Play represents a real world trial, where interventions are assessed in a natural context to increase uptake and applicability at an earlier stage of the research process^(11)^. A concurrent triangulation of the quantitative and qualitative data was integrated during the interpretation phase^(28)^. Evaluation of Appetite to Play used a knowledge exchange model, where practitioners and policy makers weighed in on the decision making process, and it was developed along with the implementation strategy^(11)^. This process was informed by the implementation evaluation literature^(29)^ and the RE-AIM framework^(30)^. RE-AIM was designed to evaluate community-based interventions and provides evaluation of outcomes at the staff level, which was undertaken in Appetite to Play^(30)^. We included satisfaction and context in our evaluation and defined maintenance as ‘potential maintenance and sustainability’. Sample size was not calculated a priori, as the goal was to disseminate at scale, reaching a large population. Implementation goals were to carry out 200 in-person workshops, connect with 3000 early years’ providers during the first 18 months and reach 1000 early years providers through the e-learning modules.

### Data collection

Participants were recruited from three groups: individuals who completed an in-person training workshop, a live-virtual workshop or a self-paced e-learning module on healthy eating. Pre- and post face-to-face workshop survey data were collected using paper-based questionnaires linked via a unique participant code. Pre and post live-online and e-learning workshops were collected anonymously through Research Electronic Data Capture (REDCap, Nashville, TN, USA). REDCap is a secure, web-based software platform for building and collecting online survey data for research studies^(31,32)^. Demographic information (e.g. age, experience, role and previous training) about participants was collected via surveys. Knowledge, confidence and intention to promote, specifically related to key workshop content and messages, were measured using a 5-point Likert scale, for example ‘Circle the statement that best describes your KNOWLEDGE about the following areas: healthy beverage choices; supporting breastfeeding; communicating about food and healthy eating with families’ and ‘Circle the statement that best describes your level of CONFIDENCE in your ability to: support children to become good eaters; offer a variety of foods at meals and snacks from Canada’s Food Guide’. These questions were developed to specifically evaluate this training programme and were based on efficacy items from the behaviour change literature and previous training programmes^(26,33,34)^.

Website analytics data were collected using Matomo freeware (Matomo, London, UK) to determine reach of the website resources.

Qualitative data came from two sources: open-ended questions on post-workshop surveys and interviews with a subset of the workshop participants. Post-workshop survey questions were (1) What do you think will make it easy for you to implement the recommended practices around healthy eating for children in your programme? (2) What do you think will make it hard for you to implement the recommended practices around healthy eating for children in your program? These responses were entered into a spreadsheet verbatim. We conducted twenty-three semi-structured telephone interviews with participants who had completed the Appetite to Play workshop and indicated on their consent form they were willing to complete an interview. We attempted to contact all 106 individuals who indicated interest and most of these participants were not reached (e.g. unreturned voicemails and emails) and a small group (*n* 4) of participants indicated they were no longer interested or unavailable. There were no apparent differences in gender, job title or location between those who were interviewed and those we were unable to contact. We asked participants questions around implementation of healthy eating recommended practices, if and how the Appetite to Play capacity-building strategies supported implementation, and what barriers and facilitators existed for supporting healthy eating in the early years.

Additional qualitative data, namely interviews with partner advisory group members and lead and regional trainers, were conducted, and data were published in a previous publication^(20)^.

Paper questionnaires were paired, and scanned and data were digitised using Remark Office OMR (Gravic, Inc., Malvern, PA, USA). Electronic questionnaires were inputted into REDCap and ready for analysis immediately. Data analysis used a mixed-methods approach^(28)^, where conclusions were drawn from both qualitative and quantitative data when available.

### Quantitative data analysis

We used IBM SPSS Statistics 27 (IBM) for statistical analysis. Descriptive statistics were calculated to describe participant characteristics and satisfaction with training. Knowledge and confidence questions were computed into four variables (pre- and post-confidence and pre-and post-knowledge) after we calculated for internal consistency using Cronbach’s alpha and found it to be sufficient (α > 0·91 for all constructs). To evaluate training outcomes, we used paired *t* tests to compare change in participant knowledge and confidence prior to and following the training. We ran ANOVAs to compare differences in training modality in terms of content, satisfaction, delivery and usefulness. All statistical assumptions for test were met.

The ‘intention to promote’ construct was built on responses to two Likert-scale statements on determination and motivation, namely: ‘I am determined to promote healthy eating for children in my care over the next 2 weeks’ and ‘I am motivated to promote healthy eating for children in my care over the next 2 weeks’. We ran an ANOVA between the intention to promote constructs from each of the three workshops to determine if there were differences in intention based on delivery modality.

### Qualitative data analysis

For open-ended questions from surveys, we used frequency counts in R to generate a preliminary data analysis. Words used less than ten times and stop words were excluded from analysis^(35,36)^. We employed this approach for the two open-ended questions on the survey responses, as there were thousands of individual responses to these two qualitative questions. This provided a preliminary filter for finding the key ideas in the many responses. Responses were read and reread by a member of the research team, and meaningful categories were generated by combining the frequently stated words, with knowledge of responses.

We used Nvivo 11 (QSR International, Burlington, MA, USA) to code and sort qualitative data. Interviews were transcribed by a professional transcriber, and then one research team member coded interviews and used content analysis to find patterns in the data^(37)^. Additional members of the research team acted as critical reviewers by reviewing themes and related quotes as presented by the coding author, a strategy to enhance trustworthiness in the data through promoting critical reflection and deeper interpretation of findings^(38,39)^. We triangulated this with quantitative data to interpret the findings of both qualitative and quantitative data.

## Results

Some of the broader reach information from this study were previously published^(20)^ because of the integration of both physical activity and healthy eating in the workshops. These are summarised here with the relevant RE-AIM framework steps to action underlined alongside the relevant results.

We evaluated reach of the Appetite to Play intervention through three outcomes: website reach, training reach and adoption and participant demographics. As reported previously,^(19, p.7)^ website visits were tracked from September 2017 to March 2019, during which the website received 25 867 individual visits (96 804 page views). Web visitors were comprised of BC residents (10 %) and often were returning visitors (38 %). Visitors spent an average of 3 min, 56 s on the website and completed 4·5 actions (e.g. page clicks and downloads) per visit, and returning visitors spent 5 min, 13 s on site, and completed 5·1 actions, indicating that returning visitors engaged more deeply with the website. Website visitors primarily accessed the site via desktop computers.

Effectiveness of the intervention was quantified by improvements in participant knowledge and confidence about healthy eating and food literacy topics, which improved significantly from pre- to post-workshop (*P* < 0·001; see Table 2). Pre- to post-workshop changes in outcomes were not significantly different between workshop modality. Cronbach’s alpha for all knowledge, confidence and intention items pre- and post-workshop, in all training modalities was high (range 0·80–0·96).

**Table 2:** Differences between knowledge and confidence in healthy eating pre and post training for different training modalities

	Type of training														
	In person workshops					Live online workshops					E-learning module				
	Pre-survey		Post-survey		*P* value	Pre-survey		Post-survey		*P* value	Pre-survey		Post-survey		*P* value
	Mean	sd	Mean	sd		Mean	sd	Mean	sd		Mean	sd	Mean	sd	
Knowledge	3·7	0·8	4·2	0·6	<0·001	3·8	0·7	4·1	0·6	<0·001	3·7	0·7	4·3	0·6	<0·001
Confidence	3·8	0·8	4·3	0·6	<0·001	3·4	0·6	4·2	0·6	<0·001	3·9	0·7	4·3	0·6	<0·001

Paired *t* tests were used to compare differences.

1 being the lowest and 5 the highest on the scale, converted from responses to 5-item Likert scale questions.

Adoption of Appetite to Play was high. Of the eighty-eight regional trainers in the geographic region of BC, 56 (64 %) were active and delivered at least one in-person workshop (mean delivery 2·8 workshops (sd 2·6); range 1–11). The regional trainers delivered a total of 195 in-person workshops, training a total of 2328 participants. Of those 2328 participants, 1670 participants (71·7 %) provided consent to participate in the research evaluation and completed pre- and post-workshop surveys. The workshops took place in seventy-two municipalities; however, some individuals travelled to workshops expanding the reach to at least 97 BC municipalities, out of 162 total^(40)^. A total of ten live online workshops were held. The online workshops trained a total of 164 participants from forty-two municipalities, within and beyond BC. Of the 164 participants, 155 (94·5 %) provided consent and completed the surveys. Finally, 249 separate participants completed the Food Literacy e-learning module, of which 159 (63·9 %) provided consent and completed the surveys. In summary, a total of 2741 participants were trained about healthy eating and food literacy of which 1733 reported they were early childhood educators, over 95 % of those trained were resident of BC. This represents approximately 11 % of the early childhood educators in BC, based on Census data^(41)^. The remaining participants worked in a variety of ECEC support roles, such as ECEC licensing officers, public health professionals (e.g. dietitians, nurses) and in programmes for children that did not provide childcare, including recreation program delivery. Table 3 summarises participant characteristics. In total, twenty-three participants who completed the training were interviewed.

**Table 3:** Summary demographics of participants of the healthy eating workshops and e-learning module and qualitative interviews

	Type of training					
	In-person workshop		Live online workshop		E-Learning HE	
	*n*	%	*n*	%	*n*	%
Adoption – Number of participants trained in using ATP (*n* 2741)	2328		164		249	
Percent who participated in the evaluation (*n* 1984)	1670	71·7 %	155	94·6 %	159	63·8 %
	%		%		%	
Percent female	95·7 %		96·7 %		95·6 %	
	Mean	sd	Mean	sd	Mean	sd
Mean age (sd)	40·1	12·4	42·9	11·4	39·2	11·9
Mean years of experience in the early years (sd)	12·1	12·4	13·2	10·1	13·2	11·0
	%		%		%	
Percent early childhood educator*	61·8 %		76·8 %		67·7 %	

sd, standard deviation.

* 80 % of participants who indicated they were not early childhood educators also indicated that they had worked a minimum of 1 year in early years settings, but participants did not consistently report their current role in early years settings.

Participants were satisfied with healthy eating content and delivery of the in-person, live online and e-learning modules and indicated the content would be useful (see Table 4). Participants indicated that the content was on average somewhat new, or slightly less than somewhat new (see Table 4). As shown in Table 4, satisfaction ratings did not differ by mode of delivery. Table 4 also show participants’ intentions to promote healthy eating following the workshop to be high and to not significantly differ across training modalities.

**Table 4:** Overall satisfaction of participants with the healthy eating workshops and E-learning module and intention to use the content of the workshops

	Type of training						Group differences *P* value
Rating questions (Likert Scale 1–5*)	In-person		Live online		E-Learning HE		
	Mean	sd	Mean	sd	Mean	sd	
Workshop content was new to me	2·9	1·3	2·7	1·1	2·6	1·1	0·39
Overall satisfied with content	4·2	1·1	4·3	0·6	4·2	0·8	0·92
Overall satisfied with delivery	4·2	1·1	4·4	0·6	4·5	0·7	0·44
Training will be useful	4·3	1·2	4·1	0·8	4·0	1·0	0·02
Intention to promote healthy eating	4·3	0·7	3·8	1·2	4·0	0·7	0·89

* Numbers on Likert scale from 1 to 5, with 1 being the lowest and 5 the highest on the scale. ANOVA were used to ascertain group differences.

### Qualitative results

Qualitative results provided detailed information on the implementation and context of Appetite to Play. Four major categories were identified from the in-person workshop participant open survey responses to questions about potential facilitators (i.e. What will make it easy). These were educator behaviours (role modeling and encouragement), providing activities and opportunities to try out food, resources (such as Appetite to Play) and focusing on fruit and vegetables. Four major categories of responses also emerged from the question about barriers. These were parental support (most ECEC centres in BC do not provide food), child-specific challenges such as picky eating and/or allergies, small food budgets (for those that did provide food) and a lack of resources. Sample quotes are displayed by category in Table 5.

**Table 5:** Key categories and sample quotes from in person workshop participant cited barriers and facilitators to implementing healthy eating protocols

Facilitators	Barriers
Educator behaviours (role modelling and encouragement) *‘Being a role model and making healthy eating fun’.*	Parental support *‘Parents provide snacks so if they provided an unhealthy item there is nothing I can do’.*
Providing activities and opportunities to try out food. *‘More activities talking about food and cooking. Healthy choices’.*	Child specific challenges *‘Some children refuse what is offered to them and they don’t want to try’.*
Resources (such as Appetite to Play) *‘Appetite to play website. Canadian food guide. Resources. etc’.*	Small budgets *‘Being a non-profit society on a budget’.*
Focusing on fruit and vegetables *‘Offering different varieties of fruits and vegetables’.*	Lack of resources *‘Lack of time and resources’.*

We found similar trends in the qualitative interview data, with early years’ providers who did not provide food explaining that this presented additional challenges, particularly as parents were not always open to education or feedback around lunchbox contents, as demonstrated in the following quote. *‘I mean I have the children open their lunch boxes you know and peel and all that stuff, but sometimes I go hmm, [you] shouldn’t be really eating this. … So I have to be always careful and weigh my words to the parents, you know oh you know, today I saw her or him, looking at this person’s lunch, you know maybe next time if you don’t mind, can you send maybe this. So, it’s lots of beating around the bush.’ (Participant 12).*

Early years’ providers extended this conversation and highlighted that food security and the housing situations of families also presented a barrier for families in providing specific food choices. Early years’ providers noted they approached the subject with caution. For example, *‘We don’t want to say bad v. good, we realize that finances can be an issue with some families, and I certainly have a few families in the center that, that is the case, and that it’s a simple matter of they can’t afford healthy choices a lot of the times…. So I think that part of it has been tricky.’ (Participant 1)*. As well, participants highlighted that some of the resources were not completely appropriate for families with very low-incomes, as highlighted here: *‘the section on Eating Well on a Limited Income, someone described this section as tips for the well off, rather than for folks for whom food security is an issue. You know cook at home more often really doesn’t work if you’re living in a Single Room Occupancy … or minimize food waste. Well hm, you know shopping the perimeter, making a grocery list if your food’s only coming from the food bank. … this section was meant to be about eating well on a limited income, and so I can understand some of the criticisms about this section.’ (Participant 21).*

For participants who worked at places that provided food, time and planning were seen as key factors in success. One participant indicated that they had staff who could devote time to focus on food preparation and planning. She said, *‘you know we’re pretty confident in our kitchen and confident in our nutrition. We have a staff member here who’s a nutritionist’ (Participant 3).* For another participant, the time needed to create healthy meals and snacks was a challenge. *‘I would say more on the healthy eating, cause we do eat generally super healthy, not always as good as we could. And I find sometimes I’ll take the easier route, just because of time factors. So probably the prep and planning, I find a little more difficult … on the snacks and healthy eating’ (Participant 18).*

In general, participants expressed that they liked the Appetite to Play program, and that it aligned with their centre philosophies on healthy eating, which aligned with survey responses. One participant said, *‘I think it was good just to get a refresher, because a lot of the time, especially for preparing snacks, our managers are actually the ones who put out the schedule of what’s prepared for snack. And it was definitely interesting to see the Canada Food Guide, and see the recommendations for healthy eating, so now when I see snacks being prepared, I can think oh this is what my manager was thinking, with different food groups being put on for snack’ (Participant 17)*. Participants also felt that while Appetite to Play was not mostly new information for them, it was an excellent refresher. One participant said, *‘a good reminder for our ECE team that yes I think when most of the parents are like okay you have to finish your whole plate, but knowing the child’s own signals and the child knows that when he’s full, when he’s not. It was a good reminder for the ECE especially, because they were on the same kind of frame, and then them passing on that message to families’. (Participant 15).*

In terms of potential maintenance and sustainability, a shift to online delivery of workshops and expansion into e-learning modules was assessed. As outlined in Tables 4 and 2, very minimal differences were seen across the three modalities, indicating high potential for long-term maintenance and sustainability through enhanced cost efficiencies.

## Discussion

The early years represent an important time in the development of healthy eating behaviours. Use of ECEC (both formal and informal) during these years is increasing and children spend substantial time in ECEC^(42,43)^. Thus, early years providers are a key health intermediary to support the development of these healthy eating behaviours amongst young children. With a growing call to disseminate and evaluate interventions in real-world settings at the population level,^(11)^ we took the opportunity to evaluate the implementation and impact of Appetite to Play on the healthy eating knowledge, confidence and practices of the early years providers that were trained. Our mixed methods, RE-AIM informed evaluation suggests that this goal was achieved.

Reach and adoption were high with workshops delivered in person to over half of all BC municipalities. Previous physical activity training data in BC demonstrated slightly lower program delivery, with forty-three workshops delivered in 9 months^(26)^. When recruitment is targeted, uptake may be higher. For example, a regionally targeted intervention in the Hunter Region of New South Wales, Australia, 91 % of ECEC centres agreed to participate in a healthy eating intervention, with 251 centres participating^(44)^.

The healthy eating implementation strategies known as Appetite to Play (categorised using Proctor^(22)^ as a capacity building in the form of a variety of training (virtual and in-person and e-module training) and resources plus the dissemination of evidence-based information encompassed within the Appetite to Play web-based toolkit appear to be effective. Confidence, knowledge, and intention to promote healthy eating all increased significantly from pre- to post-training across all three training modalities. In comparison, Ward and colleagues^(45)^ found increases in self-reported early years providers’ healthy eating practices following online training but not in person training. Devine and colleagues^(46)^ also found increases in provider knowledge following their online training.

In terms of implementation, participants were highly satisfied with workshop content, delivery and usefulness. There was one difference across training modalities, with significant differences seen in usefulness of training modality, with the e-learning modules significantly different than in-person workshops; however, we are unsure if this difference is meaningful due to the relatively low differences in means across groups (4·3–4·0/5·0). Further examination across modalities is needed. Several implementation issues arose when barriers to implementation were discussed. For instance, participants regularly reported a lack of resources (both practical and time resources) and a lack of budget as barriers to implementing healthy eating in early years settings. The implementation of the recommended practices outlined in Appetite to Play also presented challenges for early years providers, as most ECEC settings in BC do not provide food; therefore, early years providers must contend with supporting parents in providing healthy food choices to their children, rather than making changes to a menu of foods they serve the children. Previous research also indicates that parents can be perceived as barriers to implementing healthy eating in ECEC settings^(47)^. Qualitative data also indicated that child specific challenges, including individual food pickiness and/or preferences and allergies, were barriers to implementing the Appetite to Play recommended practices. Conversely, early years providers viewed themselves as role models in supporting implementation of the practices, in both their own food choices, as well as the activities and games they could share with the children they work with.

As outlined in our previous publication,^(20)^ a significant maintenance plan was developed for Appetite to Play based on recommendations from the pilot and this data collection. At the time of submission of this manuscript (2023), 4 years after the cessation of data collection, the suite of Appetite to Play resources continues to grow and redevelop based on changes in the literature. Appetite to Play continues to be hosted and managed by Child Health BC at https://appetitetoplay.com/.

### Limitations and strengths

Like all studies, this one should be viewed in light of its limitations. This study was limited by the lack of a control group. Data were collected at the level of the training participant, and there are no center or child-level data to confirm the extent to which the ideas and activities taught in the training programmes were implemented or how they may have impacted child dietary habits. No direct fidelity checks of the workshops were completed. Questions to evaluate knowledge and confidence were developed pragmatically based on typically used Likert scales and questions addressing knowledge and confidence but were specifically related to training workshop content and therefore were not validated. Likert scale responses may also be subject to social desirability bias. Some caution should be used when viewing satisfaction data, as these data represent the dual content of the workshop (physical activity as well as healthy eating).

This study also had significant strengths. Design of the intervention prioritised the needs of partners and current evidence thus enhancing potential adoption. Our sample size was large, with over 1600 participants. The intervention evaluation relied on quantitative and qualitative data and data were triangulated when overlap existed between the two information sources. Lastly, the data collection and evaluation employed well-accepted approaches from both the implementation evaluation and RE-AIM literature^(11,27,29,48)^.

### Conclusion

This paper provides support that an evidence-based capacity-building intervention with a focus on training and resources can improve knowledge, confidence and intention to promote healthy eating among early years providers. These provider-level changes are promising and a step towards improving the healthy-eating environment in ECEC settings. While it is likely that the changes observed in early years providers would have an impact on centre- and child-level outcomes, further research is warranted to confirm this, particularly as not all early years providers are in a managerial role and/or responsible for policy making^(49)^. Providers felt empowered to both use new resources and activities provided through the capacity-building intervention and to act as role models. A lack of resources and budget at the delivery system level (ECEC) to support healthy eating were reported as barriers to implementation, coupled with managing parent food provision and individual child needs around food. Most ECEC settings in the jurisdiction served by Appetite to Play do not provide food service for children, and there is no publicly funded universal food service program. Capacity-building efforts that target both parents and early years providers appear important in contexts where most food eaten in ECEC is provided by parents.
